# Heparin/Collagen-REDV Modification of Expanded Polytetrafluoroethylene Improves Regional Anti-thrombosis and Reduces Foreign Body Reactions in Local Tissues

**DOI:** 10.3389/fbioe.2022.916931

**Published:** 2022-08-04

**Authors:** Yaping Shan, Gang Chen, Qiqi Shi, Jiaxi Huang, Yaping Mi, Wenbo Zhang, Huifeng Zhang, Bing Jia

**Affiliations:** Shanghai Key Laboratory of Cardiovascular Disease, Department of Cardiovascular Center, Children’s Hospital of Fudan University, Shanghai, China

**Keywords:** expanded polytetrafluoroethylene (ePTFE), surface modification, endothelialization, foreign body reaction, biocompatibility

## Abstract

Prosthetic implants of expanded polytetrafluoroethylene (ePTFE) in the cardiovascular system have a high failure rate over the long term because of thrombosis and intimal hyperplasia. Although multiple surface modification methods have been applied to improve the anti-thrombotic and *in situ* endothelialization abilities of ePTFE, none have delivered outstanding results *in vivo*. Our previous study combined heparin/collagen multilayers and REDV peptides to modify ePTFE, and the *in-vitro* results showed that modification ePTFE with heparin/collagen-REDV can promote the cytocompatibility and antiplatelet property. This study illustrated the physical change, selective endothelial cells capture ability, and *in vivo* performance in further. The physical test demonstrated that this modification improved the hydrophilicity, flexibility and strength of ePTFE. A competition experiment of co-cultured endothelial cells and vascular smooth muscle cells verified that the heparin/collagen-REDV modification had high specificity for endothelial cell capture. A rabbit animal model was constructed to evaluate the *in vivo* performance of modified ePTFE implanted in the right ventricular outflow tract. The results showed that heparin/collagen-REDV modification was safe, promoted endothelialization, and successfully achieved regional anti-thrombosis without influencing body-wide coagulation function. The pathologic manifestations and mRNA expression pattern in tissues in contact with modified ePTFE indicated that this modification method may reduce M2-type macrophage infiltration and the expression of genes related to immune and inflammatory responses. The heparin/collagen-REDV modification may lower the incidence of complications related to ePTFE implantation and has good prospects for clinical use.

## 1 Introduction

Expanded polytetrafluoroethylene (ePTFE) is a polymer that is widely used in medical devices (Goodson, 1987; Levine and Berman, 1995; Sigurdsson et al., 1995; Redbord and Hanke, 2008). Its durable physical and chemical characteristics have made ePTFE synthetic vascular grafts the cardiovascular surgeon’s first choice for the reconstruction of blood flow when lacked the autologous grafts (Cassady et al., 2014; Ambler and Twine, 2018). In Asia, ePTFE valved conduits, which comprise an ePTFE membrane sewn to an ePTFE vascular graft, have become popular in pediatric population for the reconstruction of right ventricular outflow tract (Chang et al., 2021). Although ePTFE offers great advantages for cardiovascular uses, improvements are still needed. The biggest limitation in ePTFE implantation is the high incidence of stenosis, especially for grafts with small diameters (≤6 mm) (Sasikumar et al., 2017; Yamamoto et al., 2019; Shibutani et al., 2020).

In recent years, there have been many efforts to reduce the occurrence of stenosis following ePTFE implantation, with most of the research focused on improving anti-thrombosis using a variety of techniques (Hedayati et al., 2019; Jeong et al., 2020). However, little attention has been paid to another important cause of stenosis: foreign body reaction (FBR), a type of chronic inflammatory response that occurs locally in tissues in contact with foreign materials (Anderson et al., 2008).

Heparin was a common anticoagulant which can exert activity both *in vivo* and *in vitro*, and it was proved that heparin combined with collagen to modify materials can promote the anticoagulant properties and cell proliferation (Lu et al., 2013; Ferreira et al., 2016). Arg-Glu-Asp-Val (REDV) peptides can specific recognize *α*4*β*1 integrin receptor expressed on the membrane of endothelial cells (ECs), and it was verified that modification materials of REDV peptides can improve the ECs attachment on materials (Butruk-Raszeja et al., 2016). Our previous study first combined heparin, collagen and REDV peptides to modify ePTFE, and the results showed that this modification method inhibited platelet aggregation and promoted endothelial cells adhesion *in vitro* (Shan et al., 2018). So, we hypothesized that this modification method can also improve the anti-thrombotic and *in situ* endothelization abilities *in vivo* and reduce the stenosis after ePTFE implantation. In this study, we aimed to evaluate the safety of ePTFE modified with heparin/collagen-REDV, and to further examine its anti-thrombotic and *in situ* endothelization abilities in a rabbit model. We also investigated the degree and mechanism of FBR in local tissue implanted with this modification of ePTFE using pathologic and molecular analyses.

## 2 Materials and Methods

### 2.1 Expanded Polytetrafluoroethylene Modification

The modification of ePTFE (W.L. Gore & Associates, Newark, DE, United States) with heparin, collagen and REDV peptides was carried out according to our previously published method (Shan et al., 2018). Briefly, five bilayers of heparin (4 mg/L, Aladdin Chemistry Co., Ltd., Shanghai, China) and collagen which extracted from rat tail (each at 4 mg/L, Aladdin Chemistry Co., Ltd., Shanghai, China) were fabricated onto ePTFE membranes using a layer-by-layer technique. Successful assembly was verified by Fourier-transform infrared (FTIR) spectroscopy on a Nicolet 6,700 (Thermo Electron Corp, Waltham, MA, United States). GREDVY (Gly-Arg-Glu-Asp-Val-Tyr) peptides which containing REDV (Shanghai Science Peptide Biological Technology Co., Ltd., Shanghai, China) and labeled with fluorescein isothiocyanate were then added using a chemical grafting technique; successful grafting was confirmed by observing the fluorescence density via confocal laser scanning microscopy (Leica, Wetzlar, Germany).

### 2.2 *In vitro* Study

#### 2.2.1 Water Contact Angle Test

The water contact angle (WCA) of normal (unmodified) ePTFE (N-ePTFE, *n* = 3) and modified ePTFE (M-ePTFE, *n* = 3) was measured at room temperature using a SZ10-JC2000A contact angle goniometer (Beijing Zhongxianda Technology Co., Ltd., Beijing, China). Averaged WCA values were calculated after dropping a 3-µL volume of deionized water onto the surface of each material, tested at three different places.

#### 2.2.2 Tensile Properties

N-ePTFE (*n* = 3) and M-ePTFE (*n* = 3) membranes were cut into uniform strips with a 1.5-cm × 0.4-cm cutter. Tensile tests were carried out by an Instron 5,966 (Instron, Norwood, MA, United States) equipped with a 100-N load cell at 37°C. Samples were stretched at a constant extension speed of 10 mm/min, and tensile force was applied until the materials failed. Concurrently, a stress–strain curve was recorded, and the Young’s modulus and percent elongation at break were calculated.

#### 2.2.3 Specificity of Endothelial Cells Adsorption

The vascular endothelial cells and vascular smooth muscle cells (SMCs) were obtained from pulmonary artery of rats (Shanghai Upenol Biological Technology Co., Ltd., Shanghai, China), and cultured in high-glucose Dulbecco’s modified Eagle’s medium containing 10% fetal bovine serum at 37°C in a humidified 5% CO^2^ incubator. ECs and SMCs were labeled with the fluorescent dyes CellTracker Red (CM-Dil) and CellTracker Green (5-chloromethylfluorescein diacetate), respectively (Invitrogen, Carlsbad, CA, United States). M-ePTFE (*n* = 3) and N-ePTFE (*n* = 3) membranes were fixed with circular glass molds to the bottom of Nunc glass-bottom cell culture dishes (Invitrogen); blank wells are normal cell culture dishes without ePTFE coating were set as the Control group (*n* = 3). A mixture of ECs and SMCs in equal proportions were then seeded at a final density of 2 × 10^5^ cells/well. After co-culturing for 8 h, materials adhered to the bottom of the dishes were removed, the materials were washed three times with phosphate-buffered saline and transferred to new dishes, followed by the addition of fresh culture medium for another 24 h of co-culturing. The cell numbers and types on each material were observed at 8 and 24 h via confocal laser scanning microscopy with ×20 objective lens (Leica, Wetzlar, Germany).

### 2.3 *In vivo* Study

#### 2.3.1 Animal Model

**FIGURE 1:**
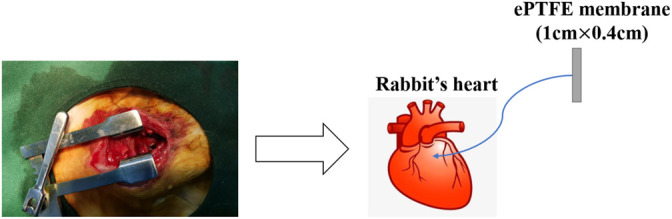
Surgical diagram of expanded polytetrafluoroethylene (ePTFE) implantation site.

We implanted M-ePTFE (*n* = 12) and N-ePTFE (*n* = 12) membranes into the right ventricular outflow tract of 3-month-old New Zealand rabbits (Shanghai Jiagan Inc., Shanghai, China). Prior to the operation, the experimental rabbits were food-fasted for 6 h, anesthetized intramuscularly with 15 mg/kg Zoletil^®^50 (Virbac S.A, France), and oxygenized with a mask. After being placed on the operating table, skin was shaved and disinfected, and a median sternal incision was made to expose the heart, and the pericardial membrane was cut to locate the right ventricular outflow tract. A purse-string suture was preset, and a 1-cm × 0.4-cm ePTFE membrane was inserted into the right ventricle from the middle of the purse-string, ensuring that the ePTFE membrane was exposed to blood and in contact with myocardium. Figure 1 showed the general surgical procedure: After fixing the ePTFE membrane in place, the sternum was closed. No anticoagulants or antiplatelet agents were used after the operation, but antibiotics were used for 3 days. Control group rabbits (*n* = 12) did not receive the operation. Body weight was measured every 2 weeks, and typical blood indexess, including inflammatory response, coagulation function, renal and liver function, and creatine kinase levels were continuously monitored at 1st, 3rd, 7th, 14th, 30th and 90th days post-operation. At 14th and 90th days postoperatively, 6 rabbits from each group were anesthetized by injecting air through an auricular vein to remove the ePTFE membranes and to collect myocardial tissue with a diameter of 0.5 cm around the implanted material. The myocardial tissue from every rabbit was divided into two parts. The first part which fixed by paraformaldehyde was used to pathologic analysis, and the second part was cut up into little pieces and stored in RNAlater (Invitrogen) at -20°C for RNA analysis. The experimental protocol was approved by the Animal Care and Ethical Committee of Children’s Hospital of Fudan University, and animal experiments were performed according to their guidelines.

#### 2.3.2 Thrombosis and Endothelialization Assessment

After removing the implanted materials from the heart and washing them with 0.9% NaCl solution, the ePTFE membrane was divided into two equal parts along the long axis; one part was fixed with 2.5% glutaraldehyde solution for observation under scanning electron microscopy (SEM, Hitachi, Tokyo, Japan), and the other was fixed with 4% paraformaldehyde solution for immunofluorescence with CD34 antibody (Cell Signaling Technology, Danvers, MA, United States). to detect ECs on the ePTFE.

#### 2.3.3 Blood Analyses

Whole blood was collected from the rabbits preoperatively, and at first, 3rd, 7th, 14th, 30th and 90th days postoperatively, for routine examinations using a BC-2800 vet automated hematology analyzer (Shenzhen Ledu Life Science and Technology Co., Ltd., Shenzhen, China). After isolating the serum, interleukin (IL)-1β was detected with an enzyme-linked immunosorbent assay kit (Invitrogen) under a BioTek ELx800 automatic microplate reader (Winooski, VT, United States). Kits were used to measure the levels of alanine aminotransferase (ALT), total bilirubin (TBIL), creatinine (Cr), and creatine kinase (CK) were measured by the corresponding kits for each using a Chemray 240 automatic biochemical analyzer (Shenzhen Reidu Life Science and Technology). To evaluate body-wide coagulation function, kits (Shenzhen Reidu Life Science and Technology) and a RAC-030 automatic blood coagulation analyzer (Shenzhen Reidu Life Science and Technology) were used to measure prothrombin time (PT), thrombin time (TT), activated partial thromboplastin time (APTT), and fibrinogen (Fib) in plasma isolated from whole blood.

#### 2.3.4 Pathologic Analysis

The myocardial tissue which fixed with paraformaldehyde were embedded with paraffin and cut into 4 μm-thick myocardial tissue. Then dewaxed it in xylene, rehydrated through decreasing concentration of ethanol. Masson trichrome staining (Wuhan Google Seville Co., Ltd., Wuhan, China) was used to make the fibrosis tissue in blue. A distant view photograph of a stained myocardium section can be obtained by PANNORAMIC scanner. Then ImageJ software (Rawak Software Inc., Stuttgart, Germany) was used to calculate the area of fibrosis by identifying blue color. The degree of inflammation was evaluated by the infiltration of M1 and M2 type macrophages in the myocardium by technique: After dewaxing and antigen recovery of myocardium tissue sections, CD86 and CD206 (Proteintech, Rosemont, IL, United States) antibodies which label M1 and M2 type macrophages respectively were incubated with sections. Then the tissue sections were incubated with secondary antibodies which labeled with purple (Alexa Fluor 647) or green (Alexa Fluor 488) fluorescence and can combined with CD86 (purple) or CD206 (green) antibodies. After that, observe the tissue section under laser scanning confocal microscope with ×20 objective lens and capture 3 pictures of each section at random. The M1 and M2 macrophage infiltration was quantized by calculated area of fluorescence color by ImageJ software.

#### 2.3.5 Transcriptome Analysis

Total RNA was extracted from myocardium surrounding the implants using a mirVana miRNA Isolation Kit (Ambion-1561; Thermo Fisher Scientific, Waltham, MA, United States) following the manufacturer’s protocol. RNA integrity was evaluated using an Agilent 2,100 Bioanalyzer (Agilent Technologies, Santa Clara, CA, United States). Samples with an RNA Integrity Number (RIN) ≥ 7 were subjected to the subsequent analyses. Libraries were constructed using a TruSeq Stranded mRNA LTSample Prep Kit (Illumina, San Diego, CA, United States) according to the manufacturer’s instructions. Transcriptome sequencing and analysis were conducted by OE Biotech Co., Ltd., (Shanghai, China). Differentially expressed mRNAs (DEmRNAs) were identified by a DESeq R package using functions to estimate size factors and run the NbinomTest (Anders and Huber). *p* values <0.05 and fold changes >2 or <0.5 were set as the threshold for significantly differential expression. The distribution of common and specific DEmRNAs in different comparison groups was illustrated by Venna analyses. To analyze the function of DEmRNAs, Gene Ontology (GO) enrichment and Kyoto Encyclopedia of Genes and Genomes (KEGG) enrichment was conducted using R studio based on the hypergeometric distribution. Each GO term can be classified according to three function domains: cellular compartment (CC), biological process (BP), and molecular function (MF); the top 30 GO terms with the smallest *p* values, or all terms if fewer than 30 met the statistical criteria, were selected for graphical display. KEGG enrichment was used to find the signal pathways which contain DEmRNAs.

### 2.4 Statistical Analysis

Continuous variables are presented as the mean ± standard deviation. The Kolmogorov-Smirnov test was used to confirm the normality of variables of each group. One-way analysis of variance was used to identify the differences between multiple groups, and the *t* test was used to identify the differences between two groups. *p* values <0.05 were considered to be statistically significant.

## 3 Results

### 3.1 Modification Expanded Polytetrafluoroethylene With Heparin/Collagen-Arg-Glu-Asp-Val

**FIGURE 2:**
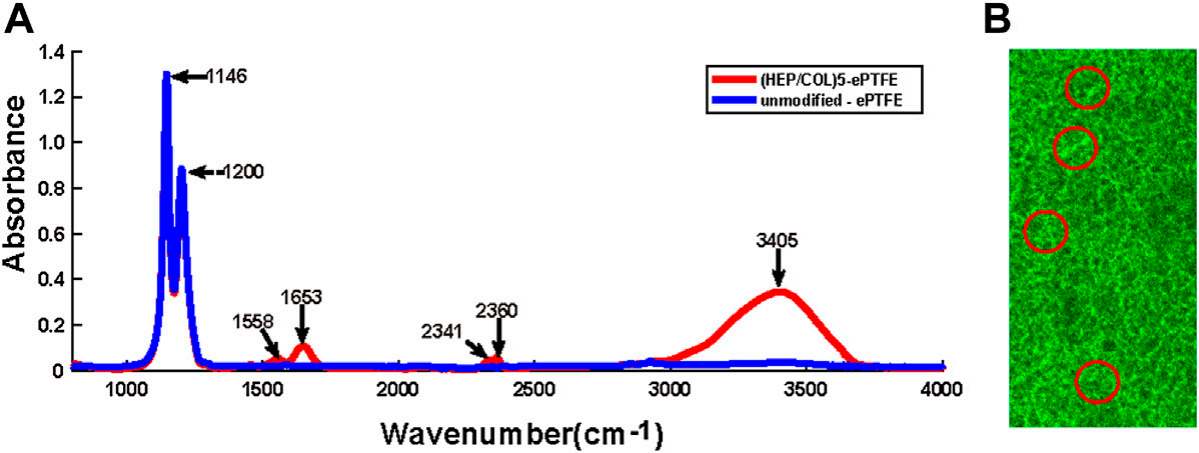
Confirmation of the assembly of heparin, collagen and REDV, Arg-Glu-Asp-Val; peptides on ePTFE, expanded polytetrafluoroethylene membranes. **(A)** Fourier-transform infrared (FTIR) spectroscopy of unmodified ePTFE and ePTFE modified with 5 bilayers of heparin and collagen (HEP/COL)5 **(B)** Confocal laser scanning microscopy illustrating the distribution of REDV peptides on modified ePTFE membranes (areas of green fluorescence circled in red color); scale bar = 75 µm.

The detection of the specific spectra of heparin (2,300–2,400 cm^−2^) and collagen (1,500–1700 cm^−2^) by FTIR spectrometry of M-ePTFE membranes indicated the successful assembly of the two substances (Figure 2A). Compact, uniform green fluorescence under confocal laser scanning microscopy confirmed the successful grafting of REDV peptides (Figure 2B).

### 3.2 *In vitro* Performance Evaluations

#### 3.2.1 WCA and Hydrophilicity

**FIGURE 3:**
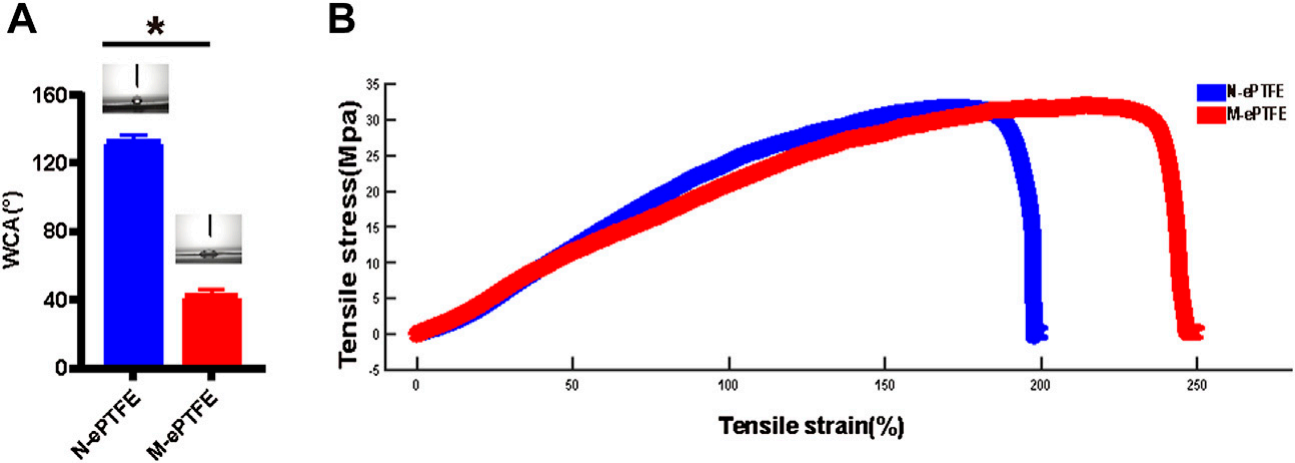
Differences in **(A)** water contact angle (WCA) and **(B)** tensile properties between unmodified N-ePTFE/M-ePTFE, expanded polytetrafluoroethylene and modified ePTFE (M-ePTFE).

As shown in Figure 3A, the average WCA of unmodified ePTFE (N-ePTFE) was 132 ± 4°, which indicated that N-ePTFE was a hydrophobic material. Upon modification with heparin/collagen-REDV, the average WCA decreased to 42 ± 4° (*p* = 0.00), demonstrating that the modification improved the hydrophilicity of ePTFE.

#### 3.2.2 Tensile Properties

As shown in Figure 3B, the heparin/collagen-REDV modification significantly decreased the Young’s modulus from 28.04 ± 0.73 MPa for N-ePTFE to 22.73 ± 0.80 MPa for M-ePTFE (*p* = 0.01), and the percent elongation increased from 217.78 ± 15.61% for N-ePTFE to 248.42 ± 10.14% for M-ePTFE (*p* = 0.044). These results indicated that the modification of ePTFE with heparin/collagen-REDV improved the flexibility of ePTFE without reducing its strength.

#### 3.2.3 Specificity of EC Adsorption

**FIGURE 4:**
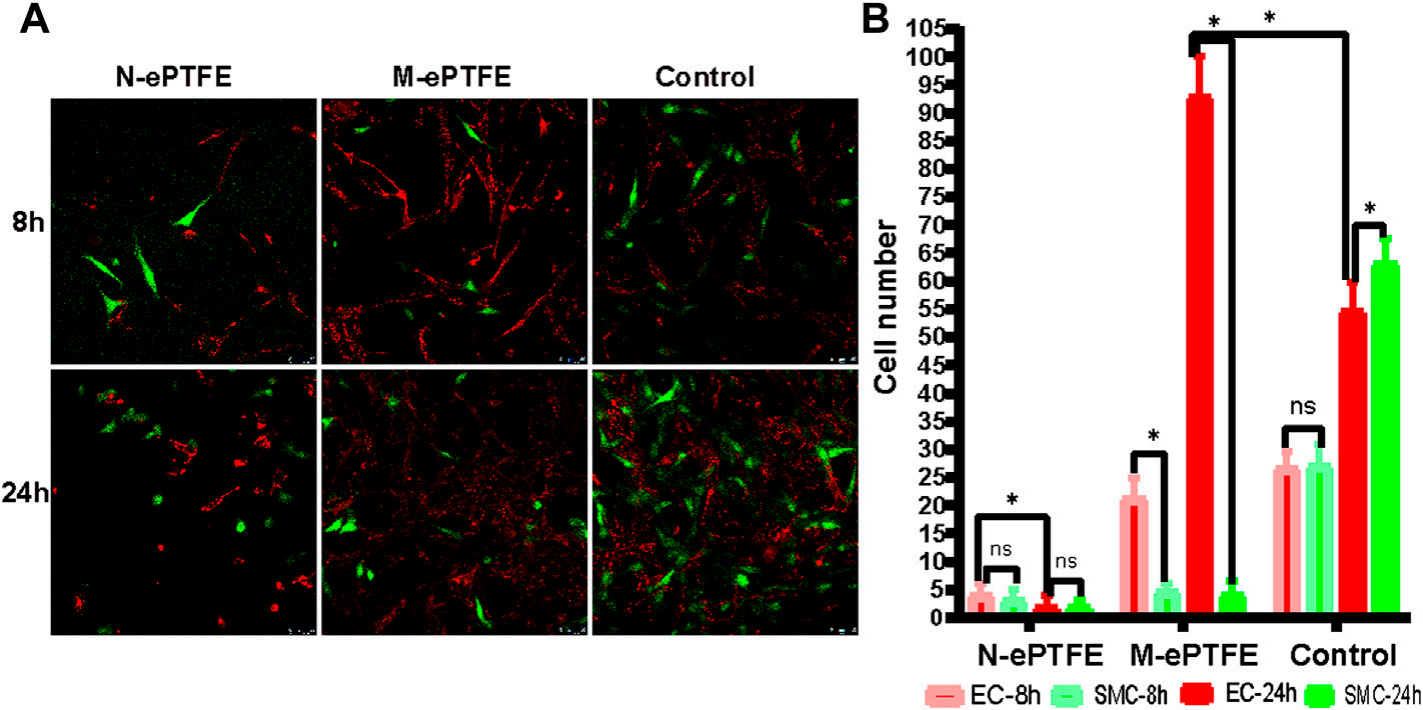
Type and number of EC, endothelial cells; and SMC, smooth muscle cells; co-cultured on unmodified N-ePTFE/M-ePTFE, expanded polytetrafluoroethylene , modified (M-ePTFE) or control plates **(A)** Distribution of EC (labeled with red dye) and SMC (labeled with green dye) in each group (scale bar = 50 μm) **(B)** Numbers of EC and SMC after co-culturing for 8 and 24 h *p < 0.05.

The distribution and the number of ECs and SMCs in different group after cultured 8 and 24 h was shown in Figure 4. Although there was no significant differences in the numbers of ECs and SMCs in the N-ePTFE (4.13 ± 2.00 vs. 3.26 ± 1.75, *p* = 0.217) and control group (26.67 ± 2.92 vs. 27.07 ± 3.79, *p* = 0.748) after 8 h co-culture, there were more ECs than SMCs in the M-ePTFE group at 8 h (21.27 ± 3.61 vs. 4.60 ± 1.64, *p* = 0.000), and the number of ECs on M-ePTFE group was larger than on N-ePTFE group (*p* = 0.000), which indicated that the modification improved ECs attachment specifically (Figure 4B). After 24 h of co-culture M-ePTFE with EC-SMC mixture, the number of ECs increased significantly (92.87 ± 7.25 vs. 21.27 ± 3.61, *p* = 0.000) and was the highest among the three groups (*p* = 0.00), the number of SMCs on M-ePTFE group at 24 h wasn’t increased when compared 8 h (4.13 ± 2.36 vs. 4.60 ± 1.64, *p* = 0.534), and the number of ECs was significantly larger than SMCs (*p* = 0.00). On Control group, both the number of ECs (26.67 ± 2.92 vs. 54.73 ± 5.08, *p* = 0.00) and SMCs (26.07 ± 3.79 vs. 63.13 ± 4.42, *p* = 0.00) increased significantly, but the number of ECs was less than SMCs (*p* = 0.00). However, both the number of ECs (4.13 ± 2.00 vs. 2.00 ± 1.89, *p* = 0.006) and SMCs (3.27 ± 1.75 vs. 1.80 ± 1.37, *p* = 0.016) decreased significantly on N-ePTFE group after 24 h co-culture. All those results indicated that the modification promoted the cytocompatibility of ePTFE and the proliferation of ECs.

### 3.3 *In vivo* Performance Evaluations

#### 3.3.1General Information on the Study Animals

In the N-ePTFE group, 17 rabbits received the operation, 3 died from surgical injuries and 2 died from diarrhea. In the M-ePTFE group, 16 rabbits received the operation, 1 died from surgical injuries and 3 died from diarrhea. In the control group, 14 rabbits received anesthesia but not the operation, and 2 rabbits died from diarrhea. All deaths happened within 1 week of the operation, and there was no significant difference in mortality rate between the N-ePTFE and M-ePTFE groups. Ultimately, 12 rabbits were included in each group; those that died were not included in the statistical analysis. The average body weights (kg) on the day of operation and 90th day later were 2.74 ± 0.12 and 3.85 ± 0.17, respectively, in the N-ePTFE group, 2.77 ± 0.95 and 4.04 ± 0.08 in the M-ePTFE group, and 2.79 ± 0.12 and 4.00 ± 0.15 in the Control group. There were no significant differences in weight at any point in time between the groups (Postoperative: *p* = 0.547, Day 90: *p* = 0.083).

#### 3.3.2 Morphologic Change and Thrombosis After Implantation

**FIGURE 5:**
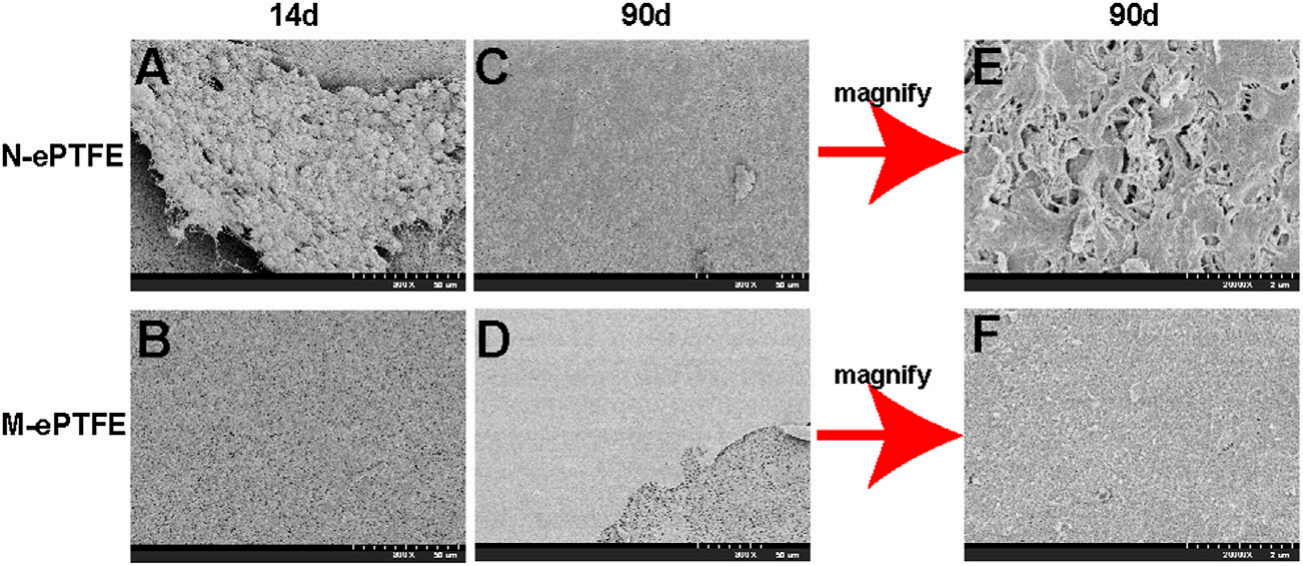
Scanning electron microscopy showing the morphology of unmodified N-ePTFE/M-ePTFE, expanded polytetrafluoroethylene and modified (M-ePTFE) at 14 d and 90 d post-implantation in rabbits. Scale bar of **(A)–(D)** = 50 μm (2000×); Scale bar of **(E,F)** = 2 μm (20000×).

At postoperative day 14, the N-ePTFE and M-ePTFE membranes that were removed from the right ventricular outflow tract were similar in appearance, with no visible changes compared with their appearance at pre-implantation (Supplementary Figure S1). Under SEM, however, there were marked differences between N-ePTFE and M-ePTFE (Figure 5A,B). There were multiple thrombi on the surface and micropores of N-ePTFE, but none on the M-ePTFE. At 90 d post-implantation, M-ePTFE membranes removed from the right ventricular outflow tract had become transparent, while N-ePTFE membranes were still white in color Supplementary Figure S1). Additionally, SEM showed that the surface of N-ePTFE was covered with a rough substance (Figure 5C) that was verified as thrombi under the higher power lens (Figure 5E). By contrast, the surface of M-ePTFE was covered with a smooth, thin film-like substance (Figure 5D) that appeared to be extracellular matrix when observed under the higher power lens (Figure 5F). There were no thrombi on the surface or micropores of M-ePTFE at 90 d post-implantation, indicating that it had good anticoagulation ability.

#### 3.3.3 Endothelialization *in vivo*

**FIGURE 6:**
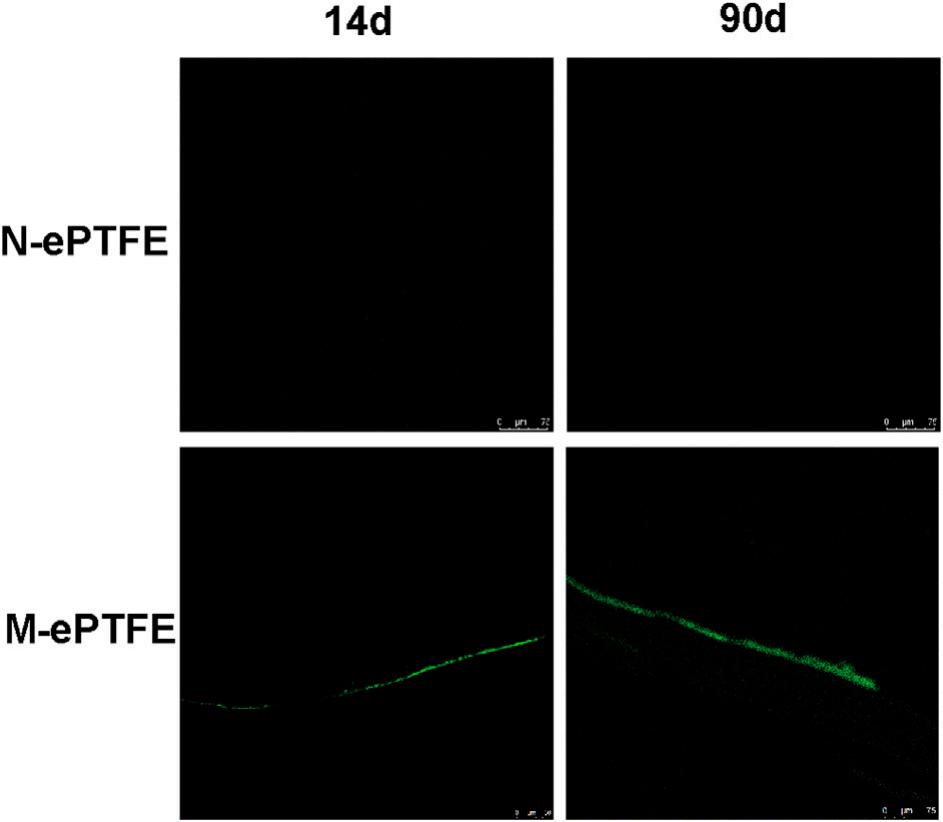
Immunofluorescence of CD34 (green) as a marker for endothelial cells on unmodified N-ePTFE/M-ePTFE, expanded polytetrafluoroethylene and modified (M-ePTFE) at 14 d and 90 d post-implantation in rabbits. Scale bar = 75 μm.

To evaluate the level of endothelialization of ePTFE membranes implanted in the rabbit body, we used immunofluorescence of CD34 antibodies as a marker for the presence of ECs. As shown at Figure 6, ECs were found on M-ePTFE both at 14d and 90 d post-implantation, and the degree of endothelization increased over time. In contrast, there was no CD34-positive cells on N-ePTFE at either time period after implantation. These results indicated that the modification of ePTFE with heparin/collagen-REDV promoted the level of endothelialization *in situ*. This endothelial barrier may potentially protect implanted M-ePTFE materials from failure.

#### 3.3.4 Systemic Inflammation Evaluation

**FIGURE 7:**
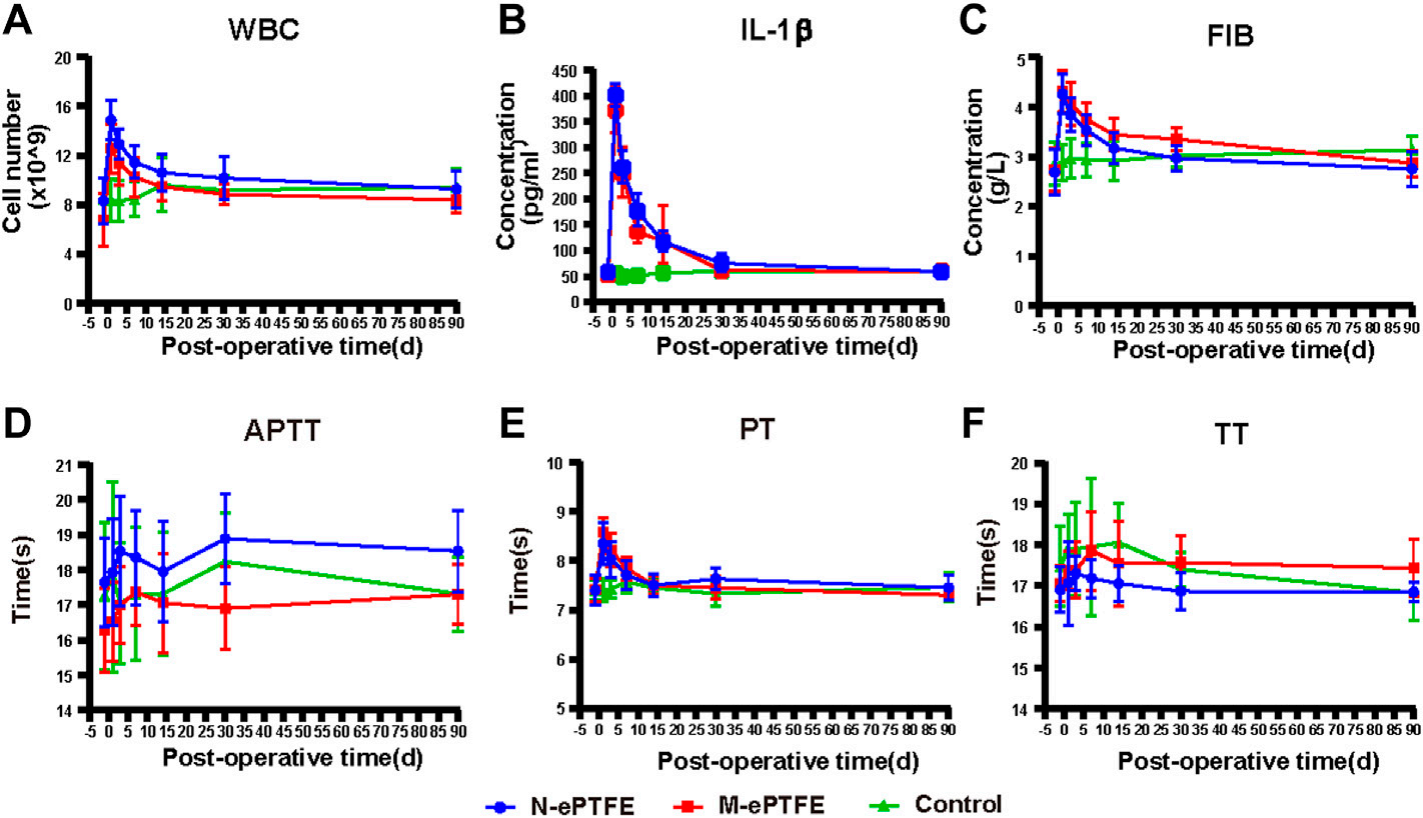
Changes in indexes for inflammation **(A–C)** and coagulation **(C–F)** in the three groups at each time point(perioperative, 1 d,3 d,7 d,14 d,30 d, 90 d after operation). WBC, white blood cells; IL-1β, interleukin-1β; FIB, fibrinogen; PT, prothrombin time; TT, thrombin time; APTT, activated partial thromboplastin time; N-ePTFE/M-ePTFE, unmodified/modified expanded polytetrafluoroethylene; Control: No implantation.

Three blood biomarkers were used to evaluate the level of body-wide inflammation: white blood cell (WBC) count, and concentrations of serum IL-1β and plasma fibrinogen. As shown in (Figures 7A–C), both the N-ePTFE and M-ePTFE implantation could lead to a significant increase of those inflammatory biomarkers when compared to control group, and the time of peak values and the time which the biomarkers recovered to preoperative level was same between N-ePTFE and M-ePTFE group, which indicated that the heparin/collagen-REDV modification did not aggravate the inflammation caused by implantation of ePTFE materials. Another finding is that the time of IL-1β returned to its preoperative level was at day 30, which was longer than the time of WBC (day 14) and fibrinogen recovered to its preoperative level.

#### 3.3.5 Systemic Coagulation Evaluation

Because the modification of ePTFE included the application of heparin, an anticoagulant, we calculated the body-wide coagulation function in each group. Although there were slight fluctuations in the three coagulation indexes (APTT, PT and TT) over time (Figures 7D–F), the there was no significant difference in the values of all those three indexes at any time point when compared to preoperative level. Those results showed that the heparin/collagen-REDV modification did not influence the body-wide coagulation function.

#### 3.3.6 Whole Body Safety Evaluation

To evaluate the safety of the heparin/collagen-REDV modification of ePTFE in rabbits, several blood indexes were continuously monitored to reflect the blood system (WBC, RBC, PLT) and hepatorenal function (ALT, TBIL, Cr). As shown at Table1, there was a decrease in the red blood cell (RBC) level in the N-ePTFE and M-ePTFE groups when compared to control group after implantation, which could be attributed to bleeding during the operation, but there was no significant difference between those two groups and it had returned to normal at 30 d postoperatively. Furthermore, there was no significant increase or decrease for other five blood indexes during the 90 d monitor in all three groups, and there were no significant differences between the three groups at every time point. All those results indicated that modification ePTFE with heparin/collagen-Arg-Glu-Asp-Val was safety.

**TABLE 1:** Blood indexess in the three study groups.

Index	Time	N-ePTFE	M-ePTFE	Control	*p* Value
RBC ( × 10^^12^/L)	preoperative	5.05 ± 0.55	5.23 ± 0.71	5.68 ± 1.71	0.380
	14 d	4.28 ± 0.63	4.28 ± 0.73	5.28 ± 0.88	0.003
	90 d	5.23 ± 0.24	5.10 ± 0.55	4.82 ± 0.81	0.467
WBC ( × 10^^9^/L)	preoperative	8.30 ± 1.89	6.76 ± 2.10	8.43 ± 1.72	0.072
	14 d	10.60 ± 1.47	9.46 ± 1.19	9.60 ± 2.20	0.206
	90 d	9.27 ± 1.50	8.37 ± 1.06	9.40 ± 1.58	0.403
PLT ( × 10^^9^/L)	preoperative	372.50 ± 200.69	293.92 ± 166.73	363.42 ± 155.80	0.494
	14 d	335.83 ± 185.57	255.48 ± 148.57	363.42 ± 152.23	0.257
	90 d	388.67 ± 188.67	329.50 ± 164.15	333.17 ± 128.46	0.788
ALT (U/L)	preoperative	47.74 ± 13.98	56.83 ± 18.35	52.00 ± 7.71	0.297
	14 d	58.80 ± 9.89	59.45 ± 13.76	54.64 ± 8.58	0.514
	90 d	51.44 ± 12.43	50.07 ± 14.04	57.10 ± 11.30	0.604
TBIL (μ*mol/L*)	preoperative	11.80 ± 3.28	10.20 ± 3.31	10.09 ± 3.07	0.359
	14 d	11.86 ± 3.09	10.68 ± 3.24	10.40 ± 3.32	0.508
	90 d	11.24 ± 3.06	9.72 ± 3.11	9.47 ± 4.48	0.663
CR (μ*mol/L*)	preoperative	57.04 ± 9.08	57.09 ± 8.15	55.23 ± 9.10	0.840
	14 d	60.82 ± 10.83	62.47 ± 7.99	55.88 ± 8.59	0.205
	90 d	52.04 ± 3.17	54.97 ± 7.54	54.23 ± 5.81	0.669

N-ePTFE/M-ePTFE, unmodified/modified expanded polytetrafluoroethylene; RBC, red blood cells; WBC, white blood cells; PLT, platelets; ALT, alanine aminotransferase; TBIL, total bilirubin; CR, creatinine.

#### 3.3.7 Myocardial Injury Evaluation

##### 3.3.7.1 CK Assessment

**FIGURE 8:**
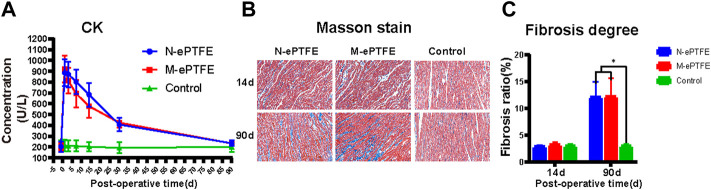
Myocardial injury evaluations **(A)** Creatine kinase (CK) concentrations in whole blood in the three groups at each time point **(B)** Masson staining of normal myocardial tissue (Control) or myocardial tissues in contact with the expanded polytetrafluoroethylene (N-ePTFE) or modified ePTFE (M-ePTFE) membranes at 14 d and 90 d post-implantation (fibrotic tissue stained in blue) **(C)** Quantification of the degree of fibrosis in the three groups at 14 d and 90 d post-implantation (*n* = 6; *p < 0.05).

CK is a typical marker used to assess the degree of myocardial injury. As shown in Figure 8A, there was no significant difference in CK before operation between three groups, but this index in N-ePTFE or M-ePTFE groups was higher than in Control groups postoperatively. However, there was no significant difference in CK between the N-ePTFE and M-ePTFE groups at any time point, and the time of CK reached a peak level or down to the perioperative level was same between two ePTFE groups, which indicated that the heparin/collagen-REDV modification did not further aggravate the myocardial injury caused by the operation itself.

##### 3.3.7.2 Degree of Myocardial Fibrosis

As shown at Figure 8B,C although fibrosis of myocardial tissue in contact with the M-ePTFE and N-ePTFE membranes did not become obvious when compared to control group at 14 d post-implantation (2.9 ± 0.3 vs. 3.2 ± 0.5 vs. 3.0 ± 0.4, *p* = 0.574)), both the degree of fibrosis in N-ePTFE or M-ePTFE group was higher than control group at 90 d (12.0 ± 2.9 vs. 12.2 ± 3.4 vs. 3.1 ± 0.5, *p* = 0.00). However, there was no significant difference between N-ePTFE and M-ePTFE group both at 14d and 90 d postoperatively. These results indicated that, although the long-term implantation of ePTFE materials in the myocardium did cause myocardial fibrosis, the heparin/collagen-REDV modification did not appear to affect the degree of fibrosis.

##### 3.3.7.3 Inflammation of the Myocardium

**FIGURE 9:**
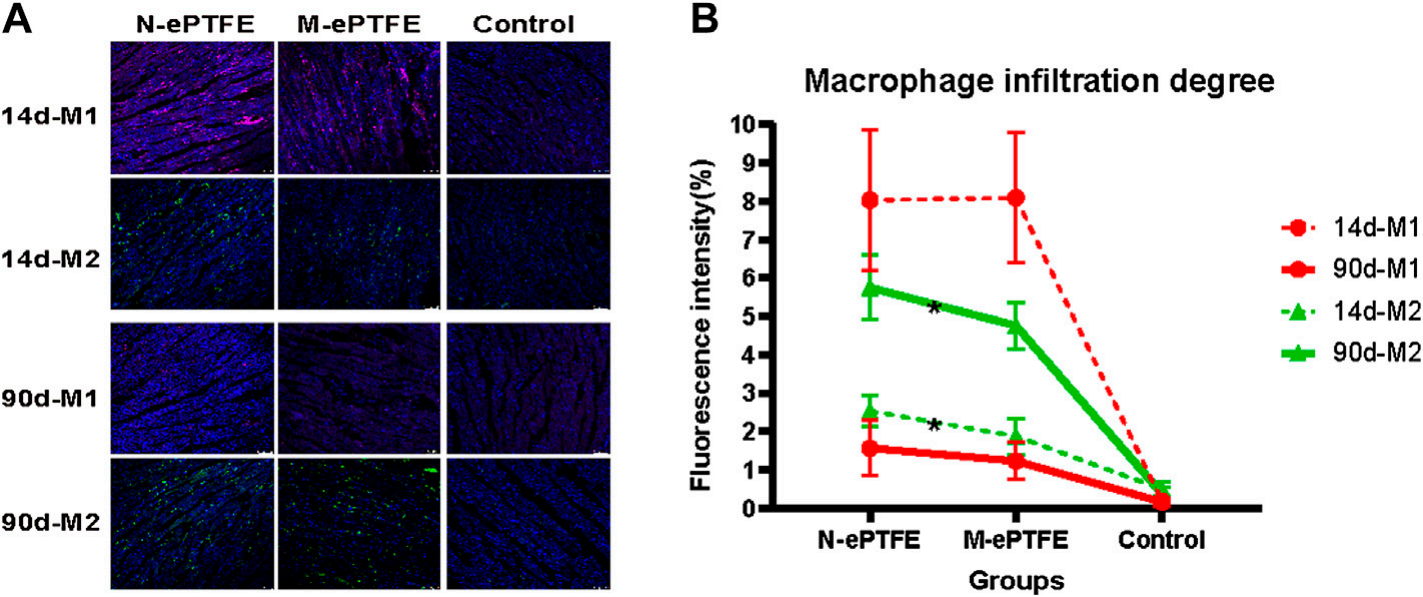
Macrophage infiltration in the myocardium **(A)** Immunofluorescence showing distribution of M1 and M2 type macrophages in the myocardium in the three groups: purple, CD86- labeled M1; green, CD206-labeled M2; blue, nuclei **(B)** Quantification of the degrees of M1 and M2 infiltration at postoperative days 14 and 90 (*n* = 6; *p < 0.05). N-ePTFE/M-ePTFE, unmodified/modified expanded polytetrafluoroethylene; Control: No implantation.

Macrophages are an important cell type involved in the inflammatory response. On the 14 d postoperatively (Figure 9A), there was increased infiltration of both M1 and M2 type macrophages in the myocardium in the N-ePTFE and M-ePTFE groups compared with that in the Control group, with more severe infiltration of M1. At 90 d after operation (Figure 9B), although there was no significant difference in the degree of M1 infiltration between the N-ePTFE and M-ePTFE groups (4.0 ± 1.4 vs. 3.3 ± 0.8, *p* = 0.085), there was a difference in M2 infiltration (2.2 ± 0.3 vs. 1.9 ± 0.5, *p* = 0.035). M1 and M2 infiltration was still apparent at 90 d after operation, but the degree of M1 infiltration in both the M-ePTFE and N-ePTFE groups was decreased compared with that at 14d, while the degree of M2 infiltration was increased in both groups (Figure 9B). The degree of M2 infiltration in the N-ePTFE group remained greater than that in the M-ePTFE group at 90 d postoperatively (5.2 ± 0.6 vs. 4.5 ± 0.7, *p* = 0.004).

##### 3.3.7.4 Altered mRNA Expression in the Myocardium

**FIGURE 10:**
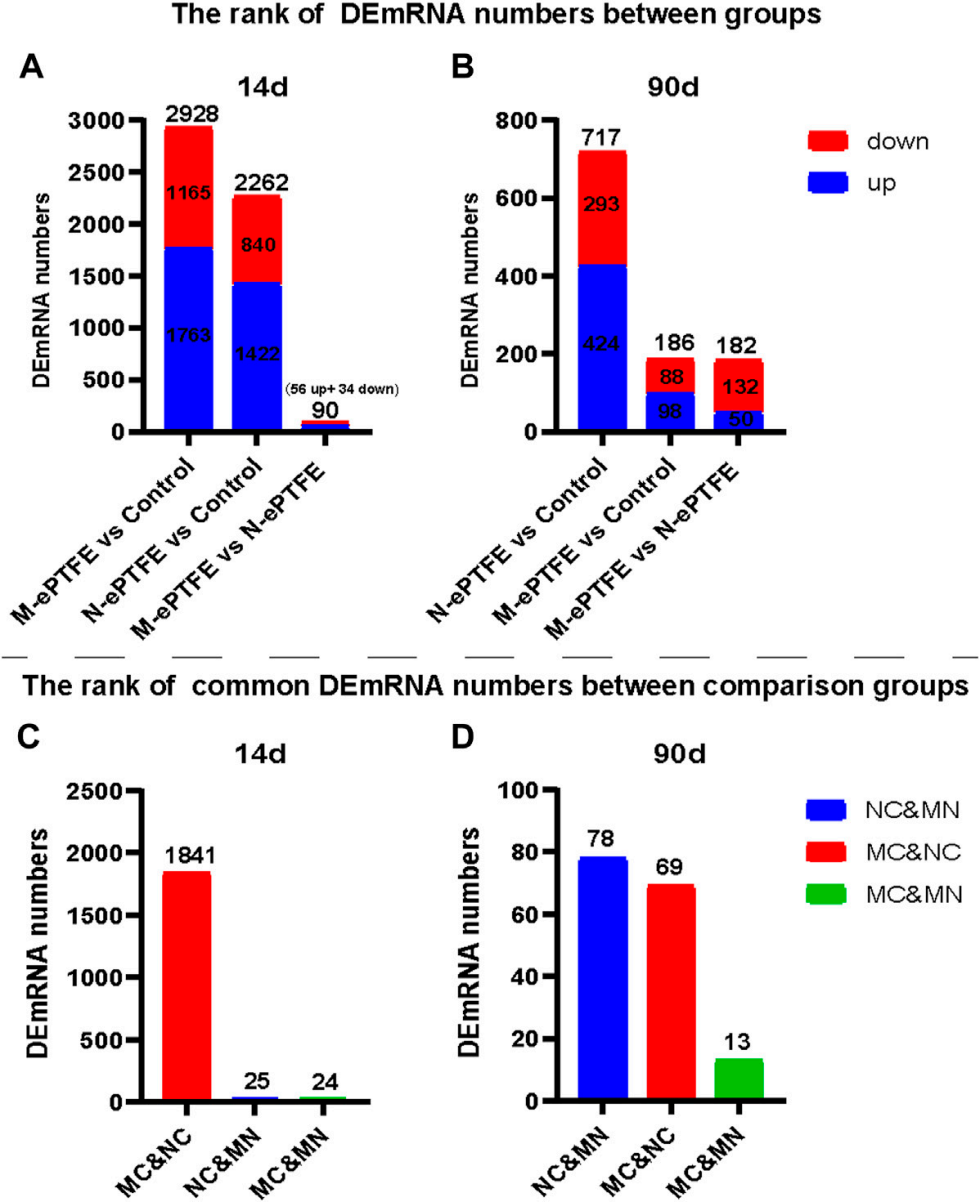
Ranking of differentially expressed mRNA (DEmRNA) in the three groups. Total numbers of DEmRNAs **(A,B)** and numbers of common DEmRNAs **(C,D)** in different group comparisons: NC, unmodified expanded polytetrafluoroethylene (N-ePTFE) vs Control comparison group; MC, modified (M-ePTFE) vs Control comparison group; MN: M-ePTFE vs N-ePTFE comparison group.

The mRNA sequencing data from myocardial tissues taken at 14 d post-implantation (Figure 10A,C) in the three groups revealed the following: the largest difference in the number of DEmRNAs was in the M-ePTFE vs. Control (MC) comparison group (2,928), followed by the N-ePTFE vs. Control (NC) comparison group (2,262); and the number of DEmRNAs in the M-ePTFE vs. N-ePTFE comparison group (MN) was smallest (90). Furthermore, the number of common DEmRNAs between different comparison groups was highest in MC and NC comparison groups. These data indicated a large degree of commonality in gene expression in tissue surrounding the M-ePTFE and N-ePTFE implants at 14 d post-implantation. At 90 d postoperatively, the gene expression patterns had changed dramatically (Figure 10B,D): the largest difference in the number of DEmRNAs was not between M-ePTFE vs. Control 186) comparison group but between the N-ePTFE and Control groups (717), which indicated that heparin/collagen-REDV modification can shorten the difference in gene expression between normal myocardium and ePTFE contacted myocardium. In addition, the comparison groups with highest common DEmRNAs were NC and MN comparison groups, which indicated that there was little similarity in gene expression patterns between the NC and MC comparison groups at 90 d when compared to 14 d. Therefore, although the heparin/collagen-REDV modification did not reduce the molecular changes in myocardium caused by ePTFE implantation in rabbits in the short term, it may be expected to reduce such changes over the long term.

**FIGURE 11:**
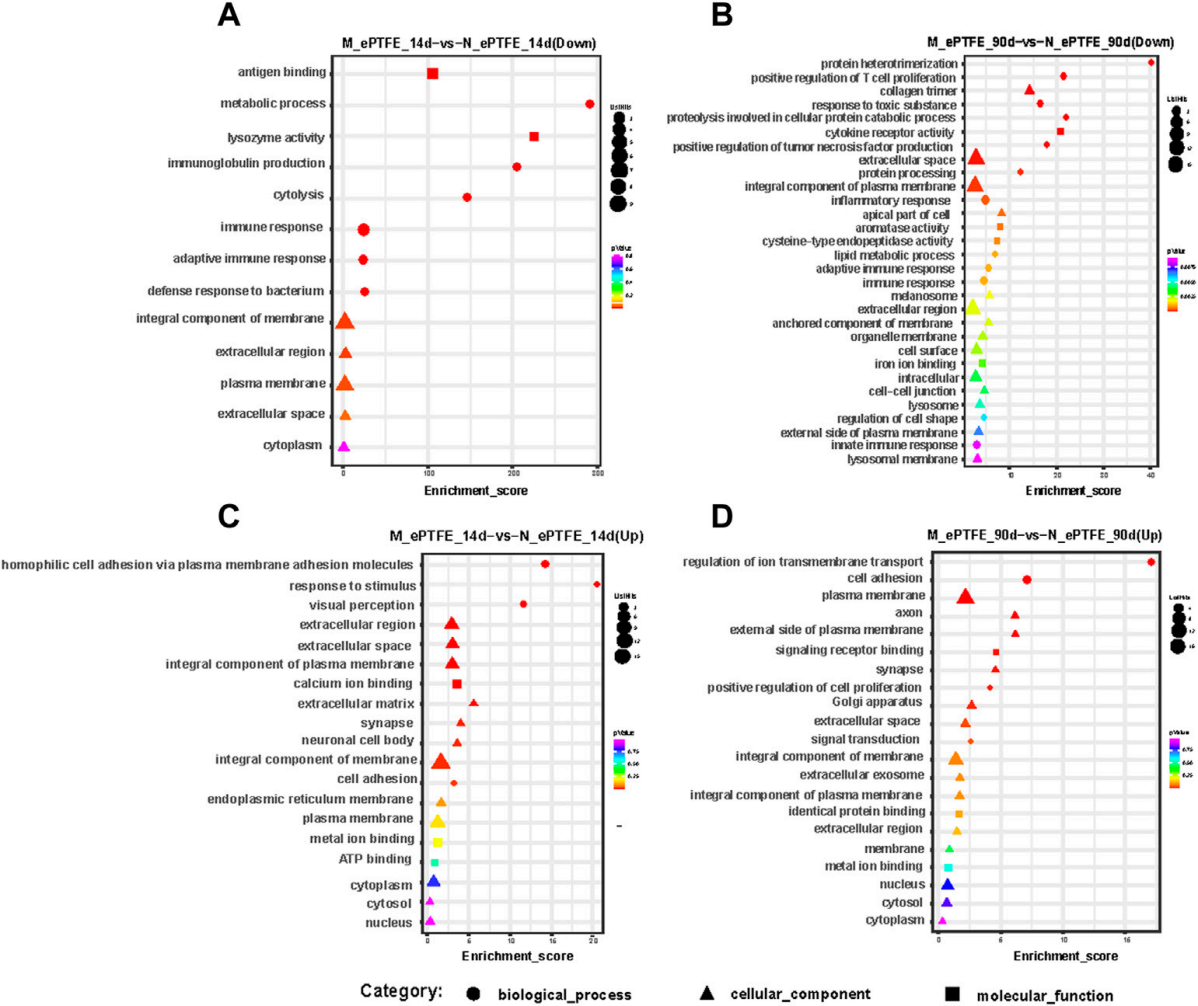
Bubble diagrams of top gene ontology (GO) terms in the modified expanded polytetrafluoroethylene (M-ePTFE) vs unmodified ePFTE (N-ePTFE) comparison group at 14 days **(A)** downregulated; **(C)** upregulated) and 90 days **(B)** downregulated, **(D)** upregulated) post-implantation. The bubble shapes indicate different categories of GO terms, while the color and diameter indicate *p* values and numbers of differentially expressed mRNAs, respectively.

Next, we analyzed the function of DEmRNAs in the MN comparison group to predict the molecular mechanisms involved in myocardial changes in response to M-ePTFE implantation. At 14 d post-implantation, GO enrichment analysis showed that DEmRNAs that were downregulated (Figure 11A) and upregulated (Figure 11B) were enriched in 13 and 19 GO terms, respectively, between the M-ePTFE and N-ePTFE groups. The enriched GO terms indicated that the ePTFE modification downregulated immune or inflammatory response-related genes and upregulated genes related to cell adhesion and the extracellular matrix (Figure 11A). At 90 d post-implantation, although the number of enriched GO terms 66) for the downregulated DEmRNAs had increased in the MN comparison groups when compared to 14d, the GO categories were still related to immune and inflammatory responses (Figure 11C). Otherwise, there were no significant changes in the total number 21) and categories of enriched GO terms among the upregulated DEmRNAs (Figure 11D). The KEGG enrichment analysis showed that there was 1 pathway related to inflammatory response with significant different between M-ePTFE and N-ePTFE comparison group at 14 d after operation, and this pathway (Cytokine-cytokine receptor interaction) contains up-regulated DEmRNAs and down-regulated DEmRNAs. At 90 d after operation, there was 3 pathways (PI3K-Akt signaling pathway, Human papillomavirus infection, Human cytomegalovirus infection) related to inflammatory response with significant different between M-ePTFE and N-ePTFE comparison group, and all those 3 pathways just containing down-regulated DEmRNAs. Overall, the result of the GO and KEGG enrichment analysis indicated that modification of ePTFE with heparin/collagen-REDV may reduce the expression of genes related to immune or inflammatory responses in local tissue caused by ePTFE implantation, especially over the long term.

## 4 Discussion

Although the hydrophobicity of ePTFE has been shown to reduce the adsorption of plasma proteins compared with other biomedical materials (Lu et al., 2013; Gao et al., 2017), the coagulation response is nevertheless induced by ePTFE implantation in the cardiovascular system (Gao et al., 2017), necessitating long-term whole-body anticoagulant therapy to prevent clinical complications (Sasikumar et al., 2017; Zhang et al., 2018). The coagulation cascade occurs immediately in response to the implantation of foreign material in the body, with the degree of response mostly dependent on the surface features of the material (Hedayati et al., 2019). Therefore, improving the surface properties of such materials is one key to increasing their anticoagulant abilities. Surface modification can be used not only to change physical properties, but also to provide regional anticoagulant therapy via coating surface materials with anticoagulant drugs (Hoshi et al., 2013). Indeed, ePTFE modified with heparin has been proven efficacious for clinical use (Samson et al., 2016; Shibutani et al., 2020). The collagen combined with heparin was designed to further stabilize the modification system, improving its anticoagulation ability, cytocompatibility and mechanical properties (Driessen et al., 2003; Ferreira et al., 2016). Surface modification can also be used to achieve endothelialization *in situ*, providing a physical barrier in addition to regulating coagulation, platelet function, and fibrinolysis (Post et al., 2019b). Although there are multiple substances used to attract ECs to implant devices, few have been proven to be effective *in vivo* (Pang et al., 2015). There are two main reasons for this: loss of activity and attraction of cell types other than ECs, such as platelets (Zhao and Feng, 2020). Recently, the REDV peptide, which attracts ECs *via* recognition of the α4β1 integrin receptor expressed on the EC membrane, was reported as having high specificity, stability and efficiency for endothelialization (Chen et al., 2018; Mahara et al., 2020; Liu X. et al., 2021; Liu Y. et al., 2021). Therefore, we designed a modification of ePTFE that combines heparin/collagen multilayers and REDV peptides to simultaneously reduce thrombosis formation and achieve *in situ* endothelialization. Our previous research showed that heparin/collagen-REDV coating on ePTFE demonstrated good anti-platelet activity and endothelial cell compatibility *in vitro* (Shan et al., 2018). This study further confirmed the good anti-thrombosis and *in situ* endothelialization ability of modified ePTFE in a rabbit model. The monitor of blood coagulation indexes showed that this modification method had no influence to endogenous and exogenous coagulation systems of the animal, which indicated that modification of ePTFE with heparin/collagen-REDV can avoid the long-term anticoagulants medication and the complications caused by anticoagulants.

After the implantation of materials, the inflammation storm happened due to stress response, surgical tissue trauma, anesthesia and infection, which can cause the increased level of white blood cells (WBC) activate and inflammation-related protein in blood (Kohl and Deutschman, 2006). This acute inflammatory response often recovered in 1 week if the pathogen related infection didn’t exist (Kohl and Deutschman, 2006), so the acute inflammatory biomarkers in our study (WBC and fibrinogen) decreased to perioperative level in 1 week in the N-ePTFE and M-ePTFE group. The IL-1β is secreted by many cells and can increase both in the acute and chronic inflammatory response, so the time of IL-1β recovered to perioperative level was longer than WBC and fibrinogen (Lopez-Castejon and Brough, 2011). As there was no significant difference in the level of inflammatory biomarkers between N-ePTFE and M-ePTFE group, the inflammatory response occurred after M-ePTFE implantation maybe not caused by modification of ePTFE. The inflammatory cascade or immune response in local tissue triggered by implantation of materials reportedly cannot be avoided (Zhou and Groth, 2018; Vasconcelos et al., 2019; Lasola et al., 2020). This immune or inflammatory response is known as a FBR(Anderson et al., 2008; Munday et al., 2014). Regardless of the type of foreign material or where it is placed in the body, the consequence of a FBR is the same: fibrotic hyperplasia, an important factor in the development of stenosis in tissues with implants (Klopfleisch and Jung, 2017; Jeong et al., 2020). This explains why stenosis frequently occurs following ePTFE implantation, even among patients receiving anticoagulant therapy (Che Man et al., 2020). Macrophages are key participants in the FBR caused by biomaterial implantation, and the macrophage phenotype can change in response to alterations of the microenvironment (Chu et al., 2020). M1 macrophages, which secrete inflammatory cytokines such as tumor necrosis factor-α, often appear in the acute inflammatory response stage, while M2 macrophages, which secrete anti-inflammatory cytokines such as IL-10, usually appear in the chronic inflammatory response stage, promoting tissue regeneration and repair (Yunna et al., 2020). Our research showed the same results: The number of M1 macrophages was larger than M2 in the early postoperative period, and the M2 number increased in the late postoperative period. Despite the compared M1 infiltration degree in myocardial tissue between the N-ePTFE group and the M-ePTFE group, the M2 number was significantly lower in M-ePTFE group. We could not conclude whether the decline is good or bad, because appropriate M2 level can promote tissue healing, or tissue regeneration and remodeling, excessive M2 infiltration can also lead to fibrosis caused by FBR (Vasconcelos et al., 2015; Boersema et al., 2016; Araujo-Gomes et al., 2019; Quan et al., 2020). However, the ideal level of M2, or ratio of M1/M2, for tissues in contact with biomaterials has not been determined (Lebre et al., 2016; Taraballi et al., 2018; Vasconcelos et al., 2019; Chu et al., 2020; Lasola et al., 2020). So, we further detect the influence in local tissue caused by ePTFE modification at molecular level. mRNA sequencing combined with bioinformatic analysis not only offers sensitive detection of alterations in total mRNA expression in tissues in contact with biomaterials, but can also predict the meaning of the alterations (Bolger et al., 2014). As reported previously, ePTFE implantation can increase the expression of inflammatory and extracellular matrix-related genes in both blood vessel tissue and cardiac tissue (Willis et al., 2004; Kwon et al., 2018; Shan et al., 2021). Our mRNA sequence data showed that the heparin/collagen-REDV modification reduced the expression levels of immune/inflammatory response-related genes in M-ePTFE implanted rabbit cardiac tissue at both 14d and 90 d after implantation, and the number of down-regulated genes was higher at 90 d after operation, which indicated that this modification method may reduce the inflammatory response in local tissue contacted with ePTFE. ‘Cytokine-cytokine receptor interaction’ pathway engaged in multiple process including adaptive inflammatory host defenses, cell growth and tissue repair, so the both the up-regulated genes and down-regulated genes in M-ePTFE vs. N-ePTFE comparison group appeared in this pathway. At 90 d after operation, there was no inflammatory response related pathway containing up-regulated genes and 3 inflammatory response related pathways containing down-regulated genes significant enriched in M-ePTFE vs. N-ePTFE comparison group. And among the 3 pathways containing down-regulated genes, PI3K-Akt signaling pathway may play a major role. Because the downstream of many inflammatory pathways and the upstream of cell proliferation or extracellular matrix-related pathways is the PI3K-Akt pathway, and the inhibit of this pathway was reported that reduce inflammatory response (Shan et al., 2021), intimal hyperplasia tumor cell proliferation (Stark et al., 2015; Zhang et al., 2020). Another exciting finding with our modification was that it reduced the gene expression gap between normal cardiac tissue and M-ePTFE–implanted cardiac tissue at 90d, which indicated that this modification method can shorten tissue repair time. On the basis of mRNA analysis, we predicted that the M2 infiltration in myocardial tissue in contact with M-ePTFE was a protective manifestation. Although the degree of fibrosis did not differ between tissues in contact with the N-ePTFE and M-ePTFE in our study, experiments conducted for a longer time period could verify this prediction. In condition that biomaterials used as grafts to connect with vessels, the intimal hyperplasia due to proliferation or migration of vascular smooth muscle cells (VSMCs) increased incidence of stenosis (Jeong et al., 2020). The mechanism of proliferation or migration of VSMCs including disruption of the vascular endothelial cell barrier (Patel et al., 2010), inflammatory response (Che Man et al., 2020) and vascular tone alternation (Post et al., 2019a). Although we didn’t evaluate the effect of heparin/collagen-REDV modification to the behavior of VSMCs *in vivo*, the *in vitro* experiment showed the lower attraction of M-ePTFE to SMC when compared to EC, and proliferation ability of SMC on M-ePTFE was lower than the SMC on normal culture dish. And both the lower macrophage infiltration and down regulated of PI3K-Akt pathway has been verified that can inhibit intimal hyperplasia in other study. All those indicated that our modification method may not aggravate the intimal hyperplasia. Further implantation of modified ePTFE grafts in an animal model of vascular transplantation was needed to verify it.

The monitor of index of blood system, hepatorenal function and the inflammation showed the good safety of our modification method, and this medication also improved the elastic modulus and strength of ePTFE according to our mechanical tensile test, which makes ePTFE a better candidate to manufacture cardiac valve (Bernacca et al., 2002).

Considering the low regenerative potential of myocardium (Fu et al., 2020), the irreplaceable function of the heart (Soliman and Rossi, 2020), the lack of research on cardiac tissue injury caused by ePTFE implantation, and duplication of animal models, we chose to use a heart model instead of a vascular graft model in rabbits. Further studies on the implantation of M-ePTFE in small-diameter vessels are needed to evaluate the efficacy of our novel modification method. This research has also provided a novel landscape of mRNA expression in myocardial tissues in contact with ePTFE over time. In future, these data may be analyzed to determine the mechanisms of biomaterial failure in the body, and to design new biomaterial targets and modifications.

## 5 Conclusions

We successfully modified ePTFE with heparin, collagen and REDV peptides and created a rabbit model to evaluate the dynamics of its performance *in vivo*. Our research revealed that the heparin/collagen-REDV modification reduced thrombosis formation and promoted endothelization on ePTFE. This novel modification method was not only biologically safe in rabbits, but it also reduced the infiltration of M2 macrophages and the expression levels of genes related to the FBR in myocardial tissues in contact with ePTFE. Taken together, our data indicate that ePTFE modified with heparin/collagen-REDV has good potential for clinical use and may be expected to lower the incidence of complications related to biomaterial implantation.

## Data Availability

The original contributions presented in the study are included in the article/Supplementary Material, further inquiries can be directed to the corresponding authors.

## Supplementary Material

Supplementary Figure S1 The appearance of unmodified expanded polytetrafluoroethylene (N-ePTFE) and modified ePTFE (M-ePTFE) which taken from animal model after 14 days or 90 days after implantation.

## Abbreviations

ePTFE, Expanded Polytetrafluoroethylene; REDV, Arg-Glu-Asp-Val; FBR, Foreign Body Reaction; ECs, Endothelial Cells; WCA, Water Contact Angle; N-ePTFE, Normal ePTFE; M-ePTFE, Modified ePTFE; SMCs, Smooth Muscle CellsSMCs; PT, Prothrombin Time; TT, Thrombin Time; APTT, Activated Partial Thromboplastin Time; Fib, Fibrinogen; DEmRNAs, Differentially Expressed mRNAs.
