# Supplementary figures and images for: Heparin/Collagen-REDV Modification of Expanded Polytetrafluoroethylene Improves Regional Anti-thrombosis and Reduces Foreign Body Reactions in Local Tissues
Source: Front Bioeng Biotechnol. 2022 Aug 4;10:916931. doi: 10.3389/fbioe.2022.916931 (PMC9386153; doi:10.3389/fbioe.2022.916931)

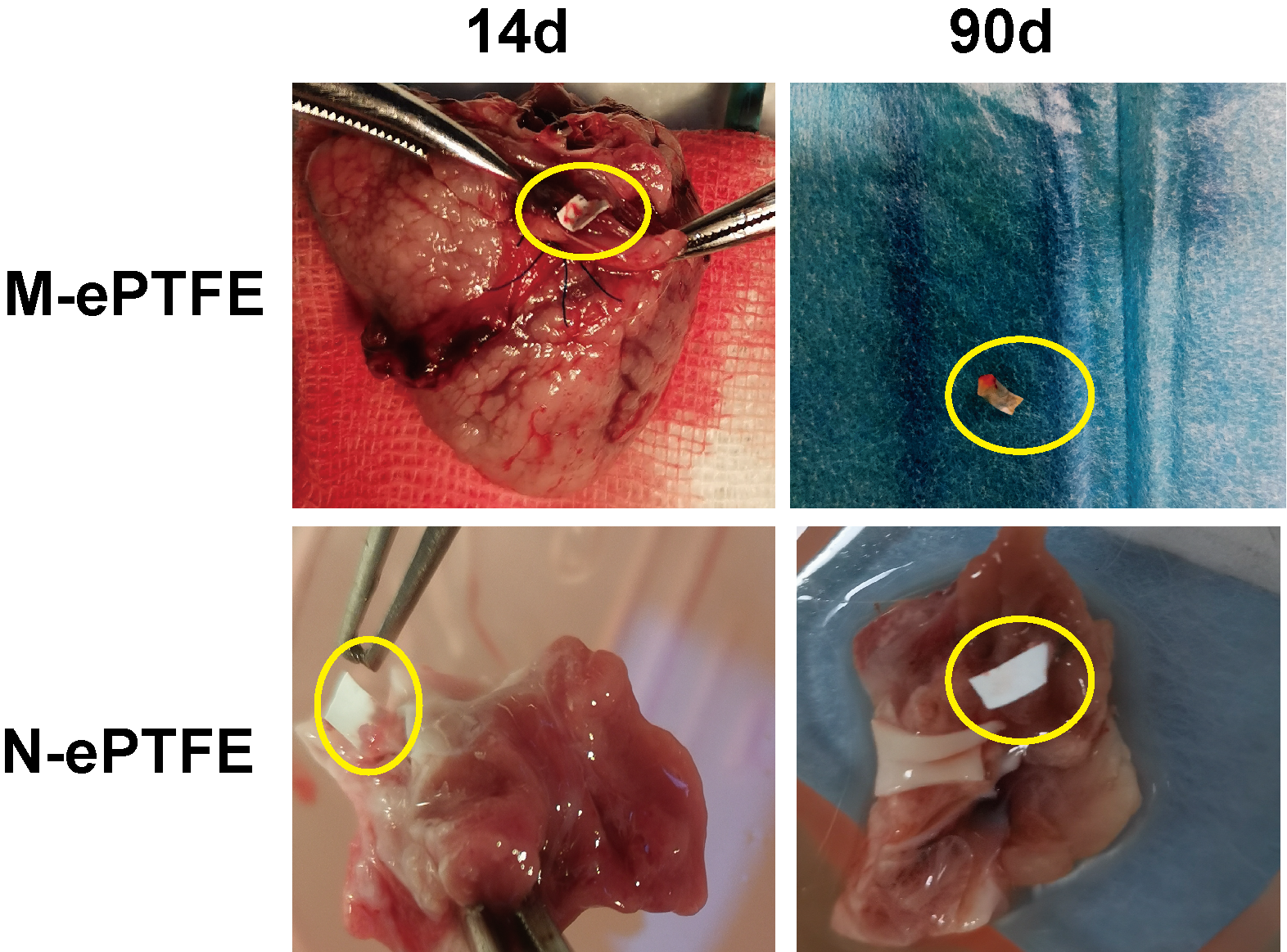

Supplement: Supplementary file 1 [file Image1.tif]
